# Role of HSP70 in response to (thermo)radiotherapy: analysis of gene expression in canine osteosarcoma cells by RNA-seq

**DOI:** 10.1038/s41598-020-69619-2

**Published:** 2020-07-29

**Authors:** Katarzyna J. Nytko, Pauline Thumser-Henner, Giancarlo Russo, Mathias S. Weyland, Carla Rohrer Bley

**Affiliations:** 1 Division of Radiation Oncology, Vetsuisse Faculty University of Zurich, 8057 Zurich, Switzerland; 2 Center for Applied Biotechnology and Molecular Medicine, University of Zurich, 8057 Zurich, Switzerland; 3 Center for Clinical Studies at the Vetsuisse Faculty of the University of Zurich, 8057 Zurich, Switzerland; 4 Functional Genomics Center Zurich, ETH/University of Zurich, Winterthurerstrasse 190, 8057 Zurich, Switzerland; 5 ZHAW School of Engineering, Zurich University of Applied Sciences, 8400 Winterthur, Switzerland; 6 BioNanomaterials Group, Adolphe Merkle Institute, University of Fribourg, Fribourg, Switzerland

**Keywords:** Bone cancer, Cancer, Radiotherapy

## Abstract

Pre-treatment of tumors with hyperthermia is often used to increase the efficacy of radiotherapy. One of the main proteins induced in response to hyperthermia is heat shock protein 70 (HSP70). The aim of our study was to investigate up- and down-regulated genes in response to (thermo)radiotherapy in HSP70 proficient and deficient canine osteosarcoma cell line (Abrams), and functional role of HSP70 in the mechanism of thermoradiosensitization. Cells were transfected with negative control siRNA or siRNA targeting HSP70 and treated with hyperthermia (HT), radiotherapy (RT), and thermoradiotherapy (HTRT). RNA sequencing was used to analyze gene expression. Hyperthermia and thermoradiotherapy, but not radiotherapy alone, induced differential gene expression. We identified genes differentially expressed only in HSP70 knockdown (thus HSP70-dependent) cells in response to hyperthermia and thermoradiotherapy. Interestingly, cell proliferation but not clonogenicity and apoptosis/necrosis was affected by the HSP70 knockdown in response to thermoradiotherapy. The results suggest that HSP70 regulates expression of specific genes in response to hyperthermia and thermoradiotherapy. Further investigations into the role of specific genes regulated in a HSP70-dependent manner in response to thermoradiotherapy could pave a way into new, combinatorial treatment options for (canine) osteosarcoma and other cancer types.

## Introduction

Radiotherapy resistance is one of the major obstacles in clinical cancer treatment of various tumor types, including osteosarcomas. Intrinsic resistance is caused by high levels of tumor hypoxia and presence of cancer stem cells, which are highly radio-resistant and are responsible for the tumor relapse after treatment^1^. Several strategies were developed to increase the efficacy of radiotherapy, including co-treatment with chemotherapeutics and also, pre-treatment with hyperthermia^2,3^. Hyperthermia is a second-line treatment modality, used mostly in refractory tumors (breast cancer, cervix carcinoma, head and neck cancer) and in combination with chemo- and radiotherapy^4^. Also in the veterinary oncology, hyperthermia has been used to treat different types of cancer (including (osteo)sarcomas)) in companion animals, in combination with radiotherapy^5,6^.

One of the main proteins induced in response to hyperthermia are heat shock proteins (HSPs)^7^. Several HSPs have been identified so far with different molecular weights and differential response to heat. Among them, HSP70 has been also shown to be overexpressed in many types of human tumors^7,8^. The role of HSP70 and other HSPs is to assist the protein folding processes and act as molecular chaperones^9^. Many regulatory proteins, including transcriptions factors, kinases and receptors, are known to be controlled by HSP70^9,10^. Therefore, HSP70 plays and important role in maintaining cellular homeostasis and can indirectly influence gene expression (for example, by regulating protein folding of transcription factors). In response to the cellular stress, heat shock transcription factor (HSF) is activated and it increases the expression of HSP70^11^. Interestingly, HSP70 interacts with HSF to negatively regulate gene expression. Moreover, HSP70 has been also showed to play an important role in posttranscriptional gene expression of selected genes^12^. Thus, apart from protecting cellular proteins during stress, HSP70 might be also directly or indirectly regulating gene expression.

We have previously shown, that Abrams canine osteosarcoma cell line was radiosensitized by hyperthermia pre-treatment^13^. Moreover, Abrams have low basal but strongly heat-inducible levels of HSP70. Therefore, we were interested in the gene expression analysis of Abrams cells in response to radiotherapy, hyperthermia and combination of both in HSP70-proficient and HSP70 knockdown Abrams cells. We used RNA sequencing technology and quantitative RT-PCR to identify down- and up-regulated factors, clonogenic cell survival and proliferation assay to measure response to treatment, and apoptosis/necrosis assay to investigate cell death after treatment.

## Methods

### Cell culture

Abrams cells were obtained from Prof. Robert Rebhun (University of California, Davis, California, USA) and cultured as described before^13^. Cells were routinely screened for Mycoplasma contamination.

### Hyperthermia treatment

Cells were treated in a humidified incubator with 5% CO_2_. The total treatment time with hyperthermia was 90 min, including the 30 min of heating phase (37–42 °C) and 60 min at 42 °C (plateau). Directly after hyperthermia (time interval between hyperthermia and irradiation was approximately 10 min), cells were transferred to radiation facility and irradiated. Prior to this study, the heating profile of the incubator used for hyperthermia treatments was characterized thoroughly by measurements of the temperature of the cell culture dish (standard 10-cm dish used for clonogenic assay)^13^. Based on data from three runs, thermal doses were calculated according to the cumulative equivalent minutes at 43 °C iso-effect model (CEM_43_)^14^. The mean thermal dose (CEM_43) was found to be 10.9 ± 0.8 min. A script written in python was used to implement the following calculation:

$${\text{CEM}}_{43} = \mathop \sum \limits_{i = 1}^{n} t_{i} \cdot R^{{\left( {43 - T_{i} } \right)}} {\text{with }}R = \left\{ {\begin{array}{*{20}c} {0.25} & {{\text{if }}T_{i} < 43^\circ {\text{C}}} \\ {0.5 } & {{\text{otherwise}}} \\ \end{array} } \right.$$

where $t_{i}$ denotes the duration of time interval $i$, and $T_{i}$ denotes the average temperature in °C during said time interval. As indicated in Nytko et al., the measurement was acquired with a Bowman probe (SPEAG/IT’IS, Zurich, Switzerland), and acquisitions were taken once per second^13^.

### Irradiation treatment

6MV photon radiation at a dose-rate of 600MU/Min (approx. 1 Gy/min) were used, and delivered by a linear accelerator (Clinac iX, Varian Medical Systems, Palo Alto, USA). Appropriated dose build-up was ensured by penetrating layers of plexiglas and the set-up was verified dosimetrically by a medical physicist.

### siRNA transfection and treatment

**Figure 1:**
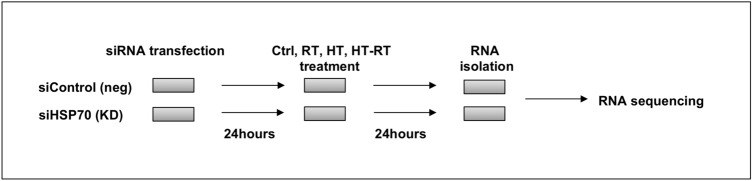
Illustration of treatment scheme of Abrams cells with hyperthermia (HT), radiotherapy (RT) and thermoradiotherapy (HTRT) before collecting RNA samples for RNA sequencing.

Cells were seeded in a 6-well plate at the density of 50,000 cells/well the day before transfection (adapted from Nytko et al.)^13^. Cells were transfected with 25 pmol of custom made siRNA against canine HSP70 (siRNAsequences in Supplementary Information 1; Thermo Fisher Scientific) and with Silencer Select Negative Control No. 1 siRNA (Cat. No. 4390843, Thermo Fisher Scientific) using Lipofectamine RNAiMAX Transfection Reagent (Thermo Fisher Scientific). 24 h after transfection medium was exchanged and cells were treated with hyperthermia, irradiation or combination of both. RNA and protein lysates samples were collected 24 h after treatment with irradiation (48 h after transfection, Fig. 1). Three independent experiments were performed resulting in a total of 24 samples (8 samples per experiment).

### RNA isolation and quantitative RT-PCR

RNA was isolated using RNeasy Mini Kit according to manufacturer’s protocol (Qiagen). Reverse transcription (RT) was performed using the iScript cDNA Synthesis Kit (BioRad) according to the manufacturer’s protocol (adapted from Ettlin et al.)^15^. KAPA PROBE FAST qPCR Kit Master Mix (2 × Universal reagents (Kapa Biosystems with 2 μL cDNA per reaction was used for quantitative polymerase chain reaction (qPCR. Samples were run in duplicates using the CFX384 Touch Real-Time PCR detection system (BioRad, Hercules, CA, US. The primers were customized Taqman gene expression assays (Thermo Fisher Scientific; Supplementary Information 1.

### RNA sequencing and analysis

The RNA sequencing was performed at the Functional Genomics Center Zürich.

### Library preparation

The method was adapted from Zatta et al.^16^. Briefly, RNA quality was tested with a Qubit (1.0) Fluorometer (Life Technologies, California, USA) and a Bioanalyzer 2100 (Agilent, Waldbronn, Germany). Samples with a 260 nm/280 nm ratio between 1.8 and 2.1 and a 28S/18S ratio between 1.5 and 2 were used for subsequent steps with TruSeq RNA Sample Prep Kit v2 (Illumina, Inc, California, USA). Total RNA samples (600 ng) were poly A enriched and then reverse-transcribed into double-stranded cDNA. The cDNA samples were fragmented, end-repaired and polyadenylated before ligation of TruSeq adapters containing the index for multiplexing. Fragments containing TruSeq adapters on both ends were selectively enriched with PCR. Qubit (1.0) Fluorometer and the Caliper GX LabChip GX (Caliper Life Sciences, Inc., USA) were used to validate the quality and quantity of the enriched libraries. The product is a smear with an average fragment size of approximately 260 bp. The libraries were normalized to 10 nM in Tris–Cl 10 mM, pH8.5 with 0.1% Tween 20.

### Cluster generation and sequencing

The method was adapted from Zatta et al.^16^. The TruSeq SR Cluster Kit HS4000 (Illumina, Inc, California, USA) was used for cluster generation using 2 nM of pooled normalized libraries on the cBOT. Sequencing were performed on the Illumina HiSeq 4000 single end 125 bp using the TruSeq SBS Kit HS4000 (Illumina, Inc, California, USA).

### Data analysis and statistics

Reads were quality-checked with FastQC. Reads at least 20 bases long, with a tail phred quality score greater than 15 and an overall average phred quality score greater than 20 were aligned to the reference genome and transcriptome (FASTA and GTF files, respectively, downloaded from the Ensembl, genome build Canis Familiaris 3.1) with STAR v.2.5.4 (1) with default settings for single end reads^17^.

Distribution of the reads across genomic isoform expression was quantified using the R package GenomicRanges (2) from Bioconductor Version 3.10^17^. Differentially expressed genes were identified using the R package edgeR (3) from Bioconductor Version 3.10^19^.

Sample clustering is shown using principal component analysis (PCA) in R version 3.6.0. For the PCA plot, we transformed the counts previously calculated using the regularized logarithm transformation or *rlog* function from DESeq2 (1.24.0) package. The heatmaps were generated using the R function *heatmap2*. Venn diagrams were created using BioVenn (http://www.biovenn.nl;^20^). Functional classification and statistical enrichment test was performed using PANTHER software (http://pantherdb.org;^21^).

### Statistics

Statistical analysis was performed using GraphPad Prism8 (GraphPad Software, Inc., San Diego, California, USA). One-column t test with Bonferroni correction was used to compare the treatment-group to the control group (set as 1), unpaired t-test was used to compare two treatment groups to each other. P values below 0.05 were considered statistically significant and denoted with a star (*), two stars (**) were used for p values below 0.01 and three stars (***) were used for p values falling below 0.001.

Immunoblotting, clonogenic, proliferation and apoptosis/necrosis assay are described in Supplementary Information 1.

## Results

### Effect of HSP70 downregulation on gene expression in Abrams cells

**Figure 2:**
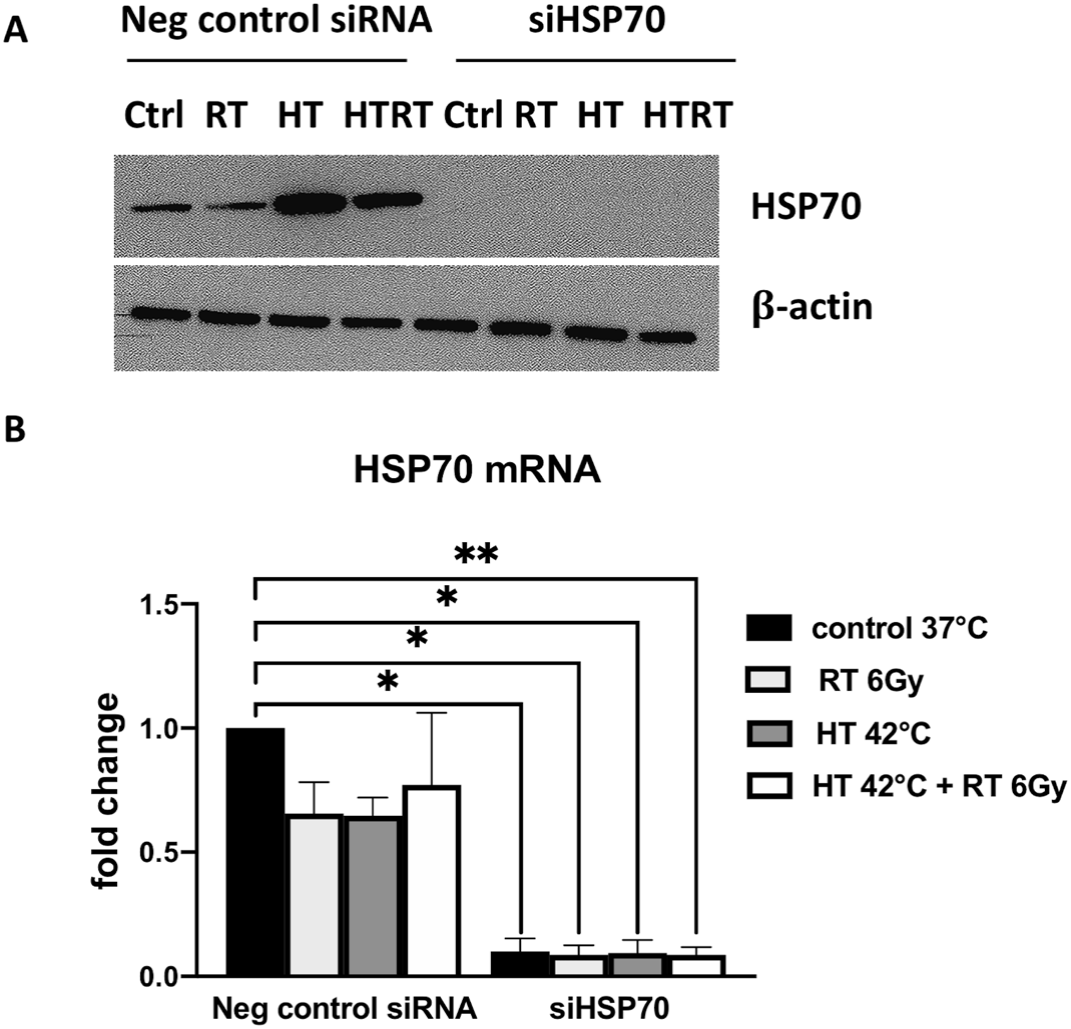
HSP70 is downregulated in osteosarcoma cells transfected with siRNA. Levels of HSP70 protein analyzed by immunoblot (**A**) and HSP70 mRNA analyzed by qRT-PCR (**B**) in negative control and HSP70 knockdown cells. Full-length blots are presented in Supplementary Fig. 3.

**Figure 3:**
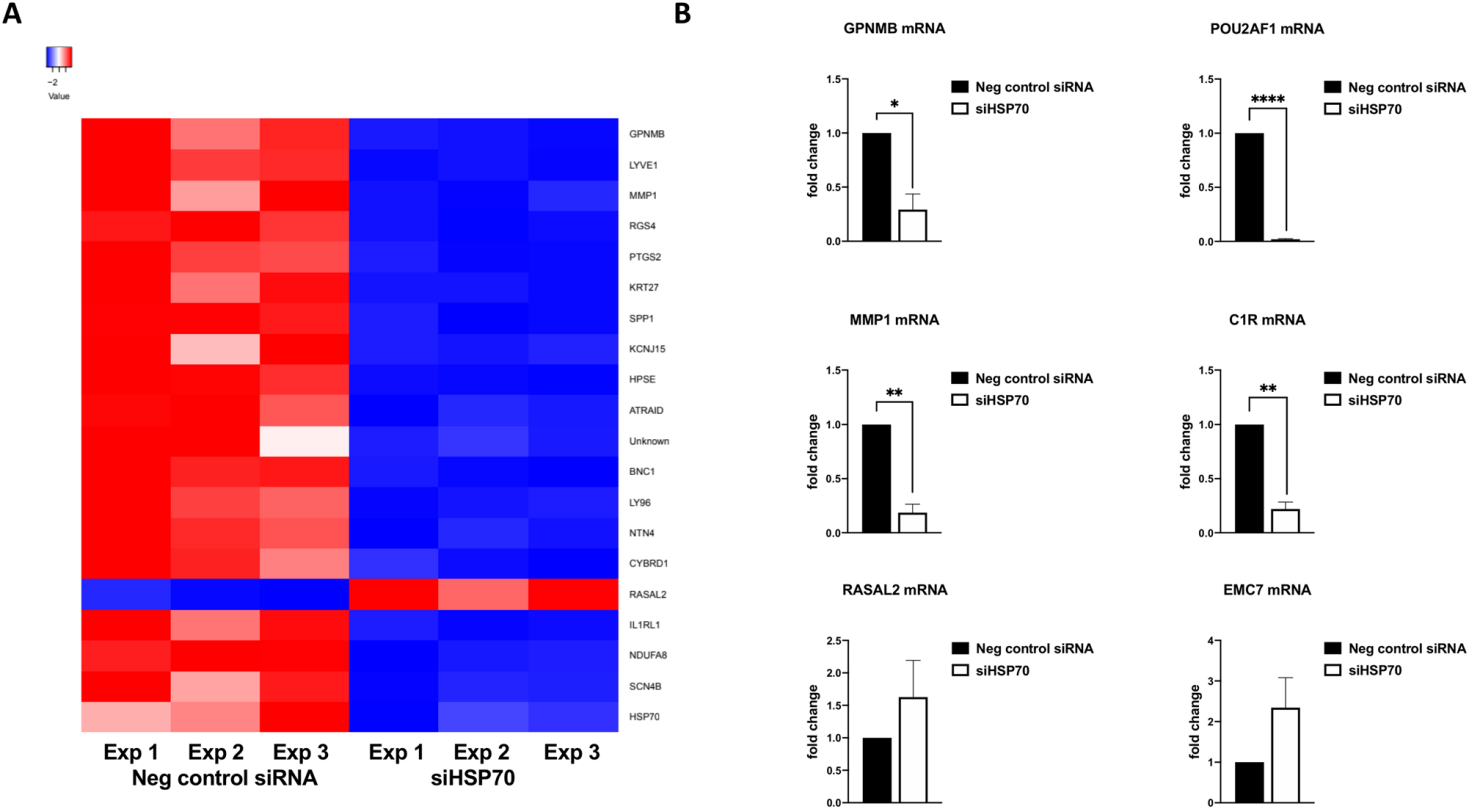
GPNMB, LYVE1 and MMP1 are the most significantly downregulated genes in HSP70 knockdown cells. Heatmap of normalized counts of the top 19 differentially expressed genes and HSP70 in negative control and HSP70 knockdown cells in experimental replicates (n = 3; **A**). mRNA levels of selected differentially expressed genes analyzed by qRT-PCR (**B**).

First, we checked the level of HSP70 protein downregulation in Abrams cells. Initially, two different siRNA sequences targeting HSP70 were tested which resulted in the same knockdown efficiency (Supplementary Fig. 1). Therefore, for the RNA sequencing experiment we proceeded only with siRNA No. 1. The protein levels of HSP70 were strongly influenced by the hyperthermia and combined treatment in negative control siRNA transfected cells but the protein was absent in control (non-treated), hyperthermia and combination-treated HSP70 knockdown cells (Fig. 2A). Additionally, we measured HSP70 mRNA, which was significantly downregulated in knockdown cells (Fig. 2B). The comparison of gene expression between Abrams cells transfected with negative control siRNA and siRNA targeting HSP70 revealed 474 differentially expressed genes (p < 0.01 and log ratio > 0.5; the 500 most significant genes in Supplementary Table 1). Functional classification according to the biological processes revealed that cellular processes, followed by the metabolic processes were the two functional categories mostly populated by the genes identified by the differential analysis (Supplementary Fig. 2A). Enrichment test revealed that the cellular component organization or biogenesis was the most significantly enriched biological process (Supplementary Fig. 2B). The top 19 differentially expressed genes and HSP70 in experimental triplicates are shown in a heatmap (Fig. 3A). The most significantly downregulated gene in the knockdown cells are glycoprotein nmb (GPNMB), followed by lymphatic vessel endothelial hyaluronan receptor 1 (LYVE1) and matrix metallopeptidase 1 (MMP1). Interestingly, among the top 20 most significantly altered genes, only two are significantly induced in the knockdown cells, namely RAS protein activator like 2 (RASAL2) and very low-density lipoprotein receptor (VLDLR). As expected, HSP70 was significantly downregulated in the knockdown cells (log2 ratio – 2.86; p = 0.000744). We confirmed the down- and up-regulation of selected genes by quantitative real-time polymerase chain reaction (qRT-PCR) in an independent experiment (Fig. 3B).

### Effects of radiotherapy, hyperthermia and thermoradiotherapy on gene expression

**Figure 4:**
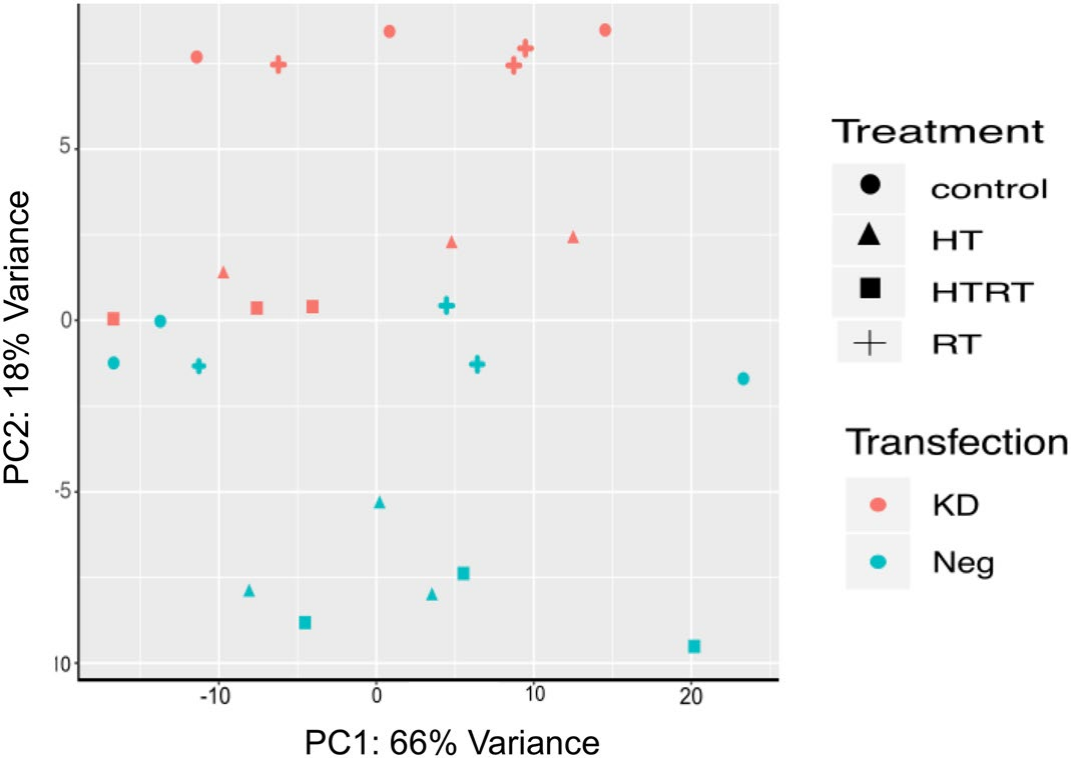
Principal component analysis score plot for all the samples used in the experiment.

Principal component analysis was performed to reveal the distances between individual samples in control and knockdown cells exposed to all treatment types (Fig. 4). It shows that negative control siRNA transfected cells and knockdown cells separate from each other, as well as treatment with hyperthermia (HT) and thermoradiotherapy (HTRT) from non-treated cells. Radiotherapy-treated cells (RT), however, do not separate from the respective non-treated cells in both, control and knockdown cells.

### Radiotherapy

In the negative control siRNA transfected cells, radiotherapy alone resulted in only 25 differentially expressed genes (p < 0.01 and log ratio > 0.5; the 500 most significant genes in Supplementary Table 2). In the HSP70 knockdown cells, irradiation alone resulted in even less, only 12 differentially expressed genes (p < 0.01 and log ratio > 0.5; Supplementary Table 3). In both comparisons, control (not-treated) over irradiated cells were compared. In both sets of comparisons, the false discovery rate (FDR) was close to 1, therefore we did not proceed with the analysis of these genes and further comparisons will focus mainly on hyperthermia- and thermoradiotherapy-induced genes.

### Hyperthermia

Hyperthermia alone resulted in 237 differentially expressed genes in negative control siRNA transfected cells and 131 in the HSP70 knockdown cells (p < 0.01 and log ratio > 0.5; the 500 most significant genes in Supplementary Tables 4 and 5), when control (non-treated) was compared to hyperthermia-treated cells.

### Thermoradiotherapy

Interestingly, the combined treatment, thermoradiotherapy, resulted in 638 differentially expressed genes in negative control siRNA transfected cells and 349 in HSP70 knockdown cells, respectively (p < 0.01 and log ratio > 0.5; the 500 most significant genes in Supplementary Tables 6 and 7), when control (not-treated) cells were compared to thermoradiotherapy-treated cells.

In general, in the HSP70 knockdown cells, fewer genes were significantly differentially expressed (for the given log ratio and significance level) than in the negative control siRNA transfected cells in response to the three treatment modalities (radiation, hyperthermia, thermoradiotherapy), with thermoradiotherapy resulting in the highest amount of the differentially expressed genes in both groups (negative control siRNA and HSP70 knockdown cells). However, when we directly compared the radiotherapy-treated cells transfected with negative control siRNA to irradiation-treated HSP70 knockdown cells, 945 differentially expressed genes were identified (p < 0.01 and log ratio > 0.5; the 500 most significant genes in Supplementary Table 8). Comparison of hyperthermia-treated negative control siRNA cells to hyperthermia-treated HSP70 knockdown cells revealed 1,367 differentially expressed genes (p < 0.01 and log ratio > 0.5; the 500 most significant genes in Supplementary Table 9). Finally, the comparison between thermoradiotherapy-treated negative control siRNA cells to thermoradiotherapy-treated HSP70 knockdown cells resulted in 3,083 differentially expressed genes (p < 0.01 and log ratio > 0.5; the 500 most significant genes in Supplementary Table 10). Many of these genes are the result of the knockdown itself. Combined treatment has the biggest impact on the gene expression in Abrams cells in comparison to single treatments, also when we directly compared the HSP70-proficient and -knockdown cells.

### Identification of HSP70-dependent genes regulated by the (thermo)radiotherapy

**Figure 5:**
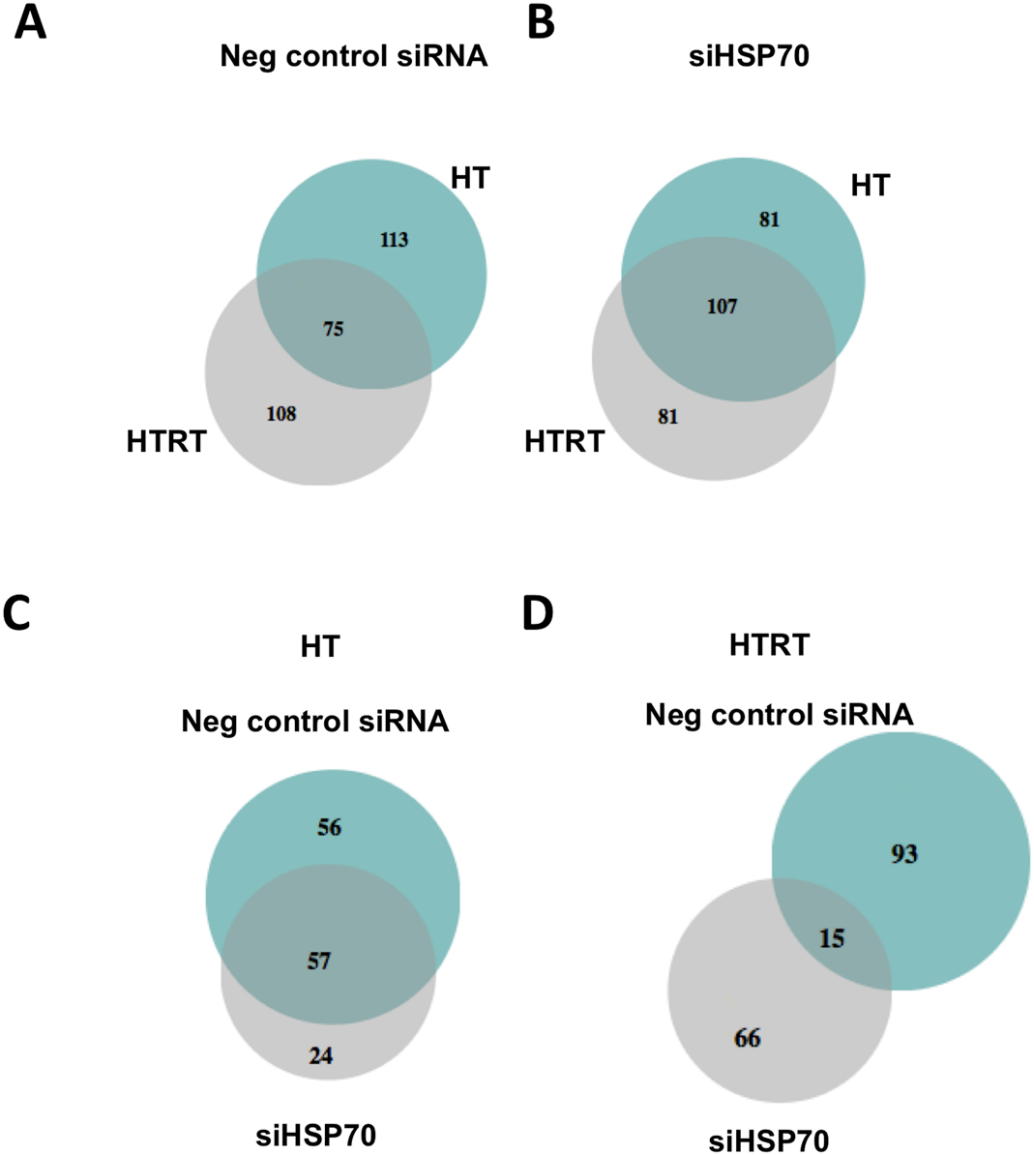
Hyperthermia and thermoradiotherapy differentially regulates the expression of certain genes in a HSP70-dependent manner. Venn diagrams showing the distribution and overlap of top 200 most significantly differentially expressed genes between the hyperthermia (HT) and thermoradiotherapy (HTRT) in negative control (**A**) and HSP70 knockdown (**B**) cells. Distribution and overlap of only hyperthermia-regulated genes (**C**, green circles in sub-figure **A** and **B**) and only thermoradiotherapy-regulated genes (**D**, grey circles in sub-figure **A** and **B**) between negative control and HSP70 knockdown cells.

In order to identify genes differentially expressed in response to thermoradiotherapy and dependent on the presence of HSP70, the Venn diagrams were created (Fig. 5). In the first comparison, the differentially expressed genes in negative control siRNA transfected cells and HSP70 knockdown cells were compared (top 200 most significantly expressed genes in hyperthermia versus non-treated and thermoradiotherapy versus non-treated, annotated genes). In negative control siRNA cells, 75 genes were common between hyperthermia and thermoradiotherapy treated cells (Fig. 5A). In the knockdown cells, 107 genes were common between the two treatment groups (Fig. 5B). As a next step, we compared the genes induced by hyperthermia only in negative control siRNA cells versus knockdown cells in order to identify genes dependent on the presence of HSP70. This comparison resulted in 24 genes differentially expressed in knockdown cells only (Fig. 5C and Supplementary Table 11). Genes involved in cellular processes were the most represented among these 24 genes (11 genes). In thermoradiotherapy-treated cells, 66 genes were differentially expressed in knockdown cells only (Fig. 5D and Supplementary Table 11). Also in this group, genes involved in cellular processes (38) were the most represented, followed by metabolic processes (13) and biological regulation (13). In the radiotherapy alone treated group (RT) only few genes were significantly induced in negative control siRNA and HSP70 knockdown cells, 25 and 12, respectively (Supplementary Tables 2 and 3). Among them (annotated genes), 2 were common between both groups, 16 were expressed in negative control siRNA cells only and 9 in knockdown cells only (Supplementary Fig. 4).

### Effect of HSP70 knockdown on cell proliferation and clonogenicity

**Figure 6:**
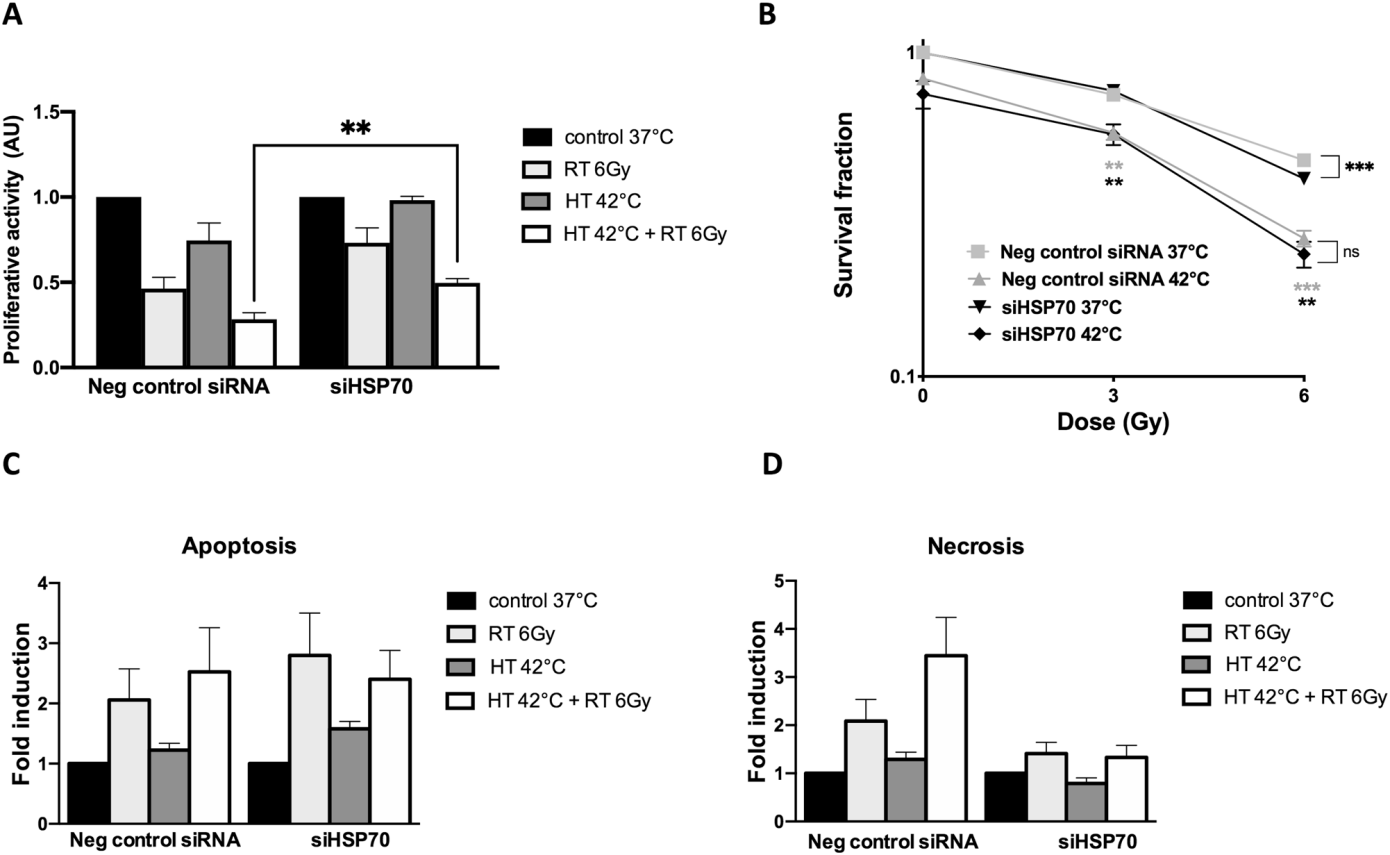
Cell proliferation but not clonogenicity and apoptosis/necrosis is affected by the HSP70 knockdown in response to thermoradiotherapy. Cell proliferation measured 96 h after treatment in negative control and HSP70 knockdown cells treated with single and combined thermoradiotherapy (**A**). Clonogenic cell survival of negative control and HSP70 knockdown after thermoradiotherapy and radiotherapy treatment (**B**). Apoptosis (**C**) and necrosis (**D**) analyzed 96 h after treatment in negative control and HSP70 knockdown cells treated with single and combined thermoradiotherapy. Mean of three independent experiments ± SEM is shown.

Negative control siRNA and HSP70 knockdown cells proliferation was inhibited by the treatment with thermoradiotherapy (Fig. 6A), the level of inhibition was significantly lower in negative control siRNA cells in comparison to the siHSP70 cells. When clonogenic cell survival of the cells was measured after treatment, both, negative control siRNA and knockdown cell clonogenicity was significantly reduced by the thermoradiotherapy treatment in comparison to the respective controls (42 °C versus 37 °C at 3 Gy and 6 Gy, Fig. 6B). Interestingly, there was significant difference between negative control siRNA cells and knockdown cells treated with 6 Gy at 37 °C but not between cells pre-treated with hyperthermia (42 °C) and treated with radiotherapy afterwards. Moreover, we have analyzed the levels of apoptosis and necrosis in negative control siRNA and HSP70 knockdown Abrams cells 96 h after treatment end. We observed small, although not significant increase in induction of apoptosis and necrosis in response to radiotherapy and thermoradiotherapy in both negative control siRNA and HSP70 knockdown cells (Fig. 6C,D).

## Discussion

Hyperthermia prior to radiotherapy is currently used in human and veterinary oncology to treat refractory tumors^22,23^. The exact mechanism of radiosensitization is in large parts explained by the increased perfusion and reoxygenation of the tumors, which leads to increased response to radiotherapy^24^. Little is known, however, about the molecular mechanism of radiosensitization at the cellular level and the role of heat shock proteins. We tried to address it by performing RNA sequencing analysis of canine osteosarcoma cells with high and low levels of HSP70 and treated with (thermo)radiotherapy. Interestingly, we found that only few genes were altered by the treatment with radiotherapy alone in both, negative control siRNA and siHSP70 osteosarcoma cells. There are very few studies that investigate gene expression after irradiation by RNA sequencing: Doan et al., showed that there were 1,094 radiation responsive genes in stable radiation-resistant glioblastoma cell line U87^25^. However, it cannot be directly compared with our study, as we looked at gene expression 24 h after irradiation with 6 Gy and in the study mentioned above, stable glioblastoma U87 cell line, generated after irradiation with 10 Gy, was used^25^. In another study, 399 and 89 genes were significantly up- or down-regulated by irradiation with 2 Gy at 6 h time point in radiation-resistant, and -sensitive prostate cancer cell lines, respectively^26^. Sarcomas are in general radiation-resistant, which could partially explain the low amount of differentially expressed genes in Abrams osteosarcoma cell line in response to radiotherapy. Moreover, it has been previously shown, that the radiation-induced gene expression is strongly dependent on the individual cell genotype, therefore the comparison with other cancer types and cell lines is not suitable^27^. In contrast, we observed that both, hyperthermia and thermoradiotherapy, induced strong changes in gene expression in HSP70-proficient and -deficient osteosarcoma cells. This is in line with another studies, where strong changes in gene expression were observed after heat shock in chicken hepatocellular carcinoma, gliomas and ovarian cancer^28–30^. Interestingly, Mahat et al., showed that gene expression changes in response to heat shock are mostly independent of heat shock factor 1 (HSF1), which regulates only fraction of heat-induced genes^31^. This could explain why we observed only 24 (out of 200 most significant) HSP70-dependent differentially expressed genes in response to hyperthermia in our study. It is also important to mention, that since we used only one siRNA in our RNA sequencing study, we cannot exclude off-target effects of the HSP70 knockdown on gene expression. Further investigations, which would include different siRNAs sequences targeting HSP70 could additionally confirm the effects we observed.

Moreover, another human and canine osteosarcoma cell lines could be used to investigate if the effects of thermoradiotherapy and HSP70 knockdown we observed are specific for this type of cancer. Interestingly, although radiotherapy alone did not induce strong gene expression changes, the combined thermoradiotherapy induced stronger changes than hyperthermia alone, suggesting, that pre-treatment with hyperthermia acts on the cellular level to sensitize cells to radiotherapy. Indeed, it has been previously shown that hyperthermia can affect DNA repair pathway in cancer cells^32^. Functionally, we observed significant changes in proliferation but not in clonogenicity between HSP70-proficient and deficient cells in response to thermoradiotherapy. Membrane-bound HSP70 has been previously shown to play an important role in the mechanism or radiosensitization^33^. Since we used HSP70 cells with low total (not only membrane-bound) HSP70 in our study, it could explain why there were no differences in clonogenic cell survival in response to thermoradiotherapy between HSP70-proficient and -deficient cells. Moreover, another studies showing the role of HSP70 in radiation sensitivity indicate, that these effects are mediated by the interaction of the surface HSP70 with immune cells and tumor microenvironment, the aspect, which we did not investigate in our study^34,35^.

In summary, knockdown of HSP70 induces gene expression changes in response to hyperthermia and thermoradiotherapy in canine osteosarcoma cell line. Further studies on the role of HSP70-dependent genes in the mechanism of thermoradiosensitization could pave a way into novel, combinatorial treatment options.

## Supplementary information

Supplementary information 1.

Supplementary information 2.

Supplementary information 3.

Supplementary information 4.

Supplementary information 5.

Supplementary information 6.

Supplementary information 7.

Supplementary information 8.

Supplementary information 9.

Supplementary information 10.

Supplementary information 11.

Supplementary information 12.

## Data Availability

The datasets generated during and/or analysed during the current study are available in the ArrayExpress repository, https://www.ebi.ac.uk/arrayexpress/experiments/E-MTAB-8652/.
