# Supplementary material for: Role of HSP70 in response to (thermo)radiotherapy: analysis of gene expression in canine osteosarcoma cells by RNA-seq
Source: Sci Rep. 2020 Jul 29;10:12779. doi: 10.1038/s41598-020-69619-2 (PMC7391659; doi:10.1038/s41598-020-69619-2)
Supplement: Supplementary file 1 — Supplementary information 1. [file 41598_2020_69619_MOESM1_ESM.docx]

**Supplementary information**

Role of HSP70 in response to (thermo)radiotherapy: analysis of gene expression in canine osteosarcoma cells by RNA-seq

Katarzyna J. Nytko^1,2,3*^, Pauline Thumser-Henner^1,2,3^, Giancarlo Russo^4^, Mathias S. Weyland^5^, Carla Rohrer Bley^1,2,3^

^1^ Division of Radiation Oncology, Vetsuisse Faculty University of Zurich, CH-8057 Zurich, Switzerland.

^2^Center for Applied Biotechnology and Molecular Medicine, University of Zurich, CH-8057 Zurich, Switzerland

^3^Center for Clinical Studies at the Vetsuisse Faculty of the University of Zurich. CH-8057 Zurich, Switzerland.

^4^Functional Genomics Center Zurich, ETH/University of Zurich, Winterthurerstrasse 190. CH-8057 Zurich, Switzerland.

^5^ ZHAW School of Engineering, Zurich University of Applied Sciences, CH-8400 Winterthur, Switzerland. BioNanomaterials Group, Adolphe Merkle Institute, University of Fribourg, Fribourg, Switzerland

^*^Corresponding author

E-mail: knytko@vetclinics.uzh.ch

**Supplementary Methods**

**Sequences of siRNAs targeting canine HSP70 (Thermo Fisher)**

| **siRNA_ID** | **sense** | **antisense** |
| --- | --- | --- |
| **No. 1** | **CCACCUACUCGGACAACCATT** | **UGGUUGUCCGAGUAGGUGGTG** |
| **No. 2** | **AGAUCGAGGUGACCUUCGATT** | **UCGAAGGUCACCUCGAUCUGG** |

**Assay ID numbers of primers used for qRT-PCR**

| **Gene symbol** | **Assay ID** |
| --- | --- |
| HSP70 | Cf02698320_g1 |
| RASAL2 | Cf02728879_m1 |
| GPNMB | Cf02632418_m1 |
| CA1 | Cf02652701_m1 |
| MMP1 | Cf02651000_g1 |
| MMP13 | Cf02741638_m1 |
| POU2AF1 | Cf02650306_m1 |
| EMC7 | Cf02635130_m1 |
| C1R | Cf02675381_m1 |

**Protein isolation and immunoblot**

Cell lysates and immunoblot were performed as described before [1]. Membranes were probed with the following antibodies: anti-HSP70 (C92F3A-5, Santa Cruz, 1:1000), beta-actin (8226, 1:1000, Abcam); and secondary anti-mouse IgG, HRP-linked Antibody (#7076, 1:2000, Cell Signaling Technology).

**Clonogenic Assay**

24 hours after transfection with siRNA, cells were trypsinized and single cell suspension was seeded in 10-centimeter dishes. The next day, cells were treated with hyperthermia, irradiation or combination of both. After treatment cells were incubated for 7 days until clones were formed and analyzed as described before [1].

**Proliferation Assay**

24 hours after transfection with siRNA, cells were trypsinized, counted and seeded in a clear 96-well plate at the density of 1’000 cells/well. The next day, cells were treated with hyperthermia, irradiation or combination of both as described above. Cell viability was measured using CCK-8 assay (Dojindo) 96 hours after treatment.

**Apoptosis and necrosis assay**

24 hours after transfection with siRNA (negative control siRNA and siHSP70), cells were trypsinized, counted and seeded in a white 96-well plate at the density of 1’000 cells/well. The next day, cells were treated with hyperthermia, irradiation or combination of both as described above. Apoptosis and necrosis were measured 96 hours after treatment using RealTime-Glo Annexin V Apoptosis and Necrosis Assay according to the manufacturer’s protocol (Promega), the assay was coupled with CellTiter-Glo 2.0 Cell Viability Assay (Promega) to normalize the values to cell viability.

Supplementary Figure 1

Supplementary Figure 2


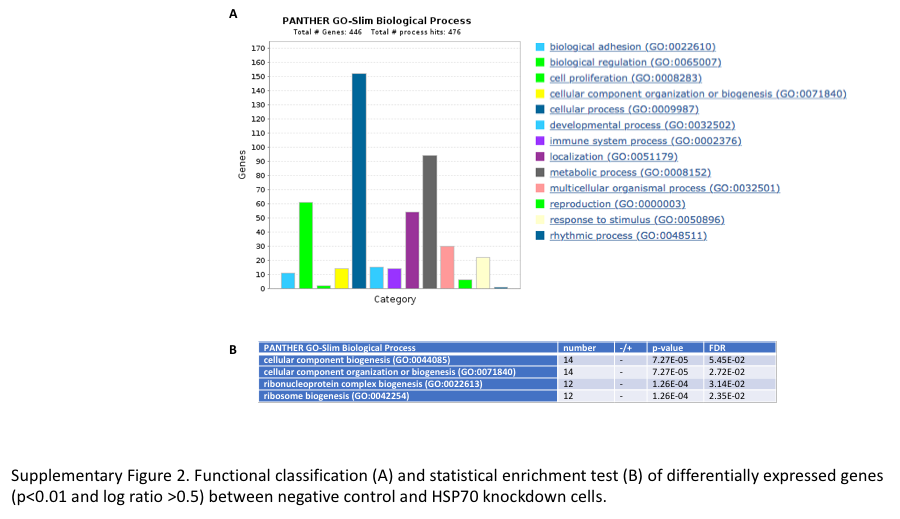


Supplementary Figure 3

Supplementary Figure 4

References:

1. Nytko, K.J., et al., *Cell line-specific efficacy of thermoradiotherapy in human and canine cancer cells in vitro.* PloS one, 2019. **14**(5): p. e0216744.
